# Impact of preoperative plasma levels of interleukin 6 and interleukin 6 soluble receptor on disease outcomes after radical cystectomy for bladder cancer

**DOI:** 10.1007/s00262-021-02953-0

**Published:** 2021-05-23

**Authors:** Victor M. Schuettfort, Benjamin Pradere, Quoc-Dien Trinh, David D’Andrea, Fahad Quhal, Hadi Mostafaei, Ekaterina Laukhtina, Keiichiro Mori, Reza Sari Motlagh, Michael Rink, Pierre I. Karakiewicz, Piotr Chlosta, Jeremy Yuen-Chun Teoh, Yair Lotan, Douglas Scherr, Mohammad Abufaraj, Marco Moschini, Shahrokh F. Shariat

**Affiliations:** 1 Department of Urology, Comprehensive Cancer Center, Vienna General Hospital, Medical University of Vienna, Währinger Gürtel 18-20, 1090 Vienna, Austria; 2 Department of Urology, University Medical Center Hamburg-Eppendorf, Hamburg, Germany; 3 Division of Urological Surgery and Center for Surgery and Public Health, Brigham and Women’s Hospital, Harvard Medical School, Boston, MA USA; 4 Department of Urology, King Fahad Specialist Hospital, Dammam, Saudi Arabia; 5 Research Center for Evidence Based Medicine, Tabriz University of Medical Sciences, Tabriz, Iran; 6 Institute for Urology and Reproductive Health, Sechenov University, Moscow, Russia; 7 Department of Urology, The Jikei University School of Medicine, Tokyo, Japan; 8 Cancer Prognostics and Health Outcomes Unit, Division of Urology, University of Montreal Health Center, Montreal, Canada; 9 Department of Urology, Medical College, Jagiellonian University, Krakow, Poland; 10 S.H. Ho Urology Centre, Department of Surgery, Prince of Wales Hospital, The Chinese University of Hong Kong, Hong Kong, China; 11 Department of Urology, University of Texas Southwestern, Dallas, TX USA; 12 Department of Urology, Weill Cornell Medical College, New York Presbyterian Hospital, New York, USA; 13 Division of Urology, Department of Special Surgery, Jordan University Hospital, The University of Jordan, Amman, Jordan; 14 Department of Urology, Luzerner Kantonsspital, Lucerne, Switzerland; 15 Department of Urology, Institut Mutualiste Montsouris, Paris, France; 16 Department of Urology and Division of Experimental Oncology, Urological Research Institute, Vita-Salute San Raffaele, Milan, Italy; 17 Department of Urology, Hospital Motol, Second Faculty of Medicine, Charles University, Praha, Czech Republic; 18 Karl Landsteiner Institute of Urology and Andrology, Vienna, Austria; 19 European Association of Urology Research Foundation, Arnhem, Netherlands

**Keywords:** MIBC, Biomarker, Bladder cancer, Interleukin 6, Interleukin 6 soluble receptor, Urothelial carcinoma

## Abstract

**Background:**

Preoperative plasma levels of Interleukin 6 (IL6) and its soluble receptor (IL6sR) have previously been associated with oncologic outcomes in urothelial carcinoma of the bladder (UCB); however, external validation in patients treated with radical cystectomy (RC) for UCB is missing.

**Patients/methods:**

We prospectively collected preoperative plasma from 1,036 consecutive patients at two institutes. These plasma specimens were assessed for levels of IL6 and IL6sR. Logistic and Cox regression analyses were used to assess the correlation of plasma levels with pathologic and survival outcomes. The additional clinical net benefits of preoperative IL6 and IL6sR were evaluated using decision curve analysis (DCA).

**Results:**

Median IL6 and IL6sR plasma levels were significantly higher in patients with adverse pathologic features. Elevated biomarker levels were independently associated with an increased risk for lymph node metastasis and ≥ pT3 disease. Both biomarkers were independently associated with recurrence-free survival (RFS), cancer-specific survival (CSS) and overall survival (OS). The addition to, respectively, fitted pre- and postoperative prognostic models improved the predictive accuracy for lymph node metastasis, ≥ pT3 disease, RFS and CSS on DCA.

**Interpretation:**

We confirmed that elevated preoperative plasma levels of IL6 and IL6sR levels are associated with worse oncological disease survival in patients treated with RC for UCB in a large multicenter study. Both biomarkers hold potential in identifying patients with adverse pathological features that may benefit from intensified/multimodal therapy and warrant inclusion into predictive/prognostic models. They demonstrated the ability to improve the discriminatory power of such models and thus guide clinical decision making.

## Introduction

Radical cystectomy (RC) is the standard of care for very high risk and Bacillus Calmette–Guerin (BCG) unresponsive non-muscle invasive bladder cancer (NIMBC) as well as muscle invasive bladder cancer (MIBC) [1–4]. Despite adequate therapy with curative intent, a significant proportion of patients are misstaged, resulting in suboptimal outcomes [5–9]. Patient selection for tailored therapy remains challenging, as we are still lacking clinically reliable biomarkers for outcome prediction [9–11]. So far, the best prognostic models are based on postoperative pathologic features, while preoperative outcome prediction remains inaccurate, although it is, at least, of equal importance [11–15]. Novel biomarkers that are readily available and sufficiently improve current predictive and prognostic models for outcome prediction are desperately needed, in order to accurately predict the clinical behavior of each tumor in each patient at that time [10, 11, 16, 17].

As pro-inflammatory cytokines were found to be essential in the pathogenesis of urothelial carcinoma of the bladder (UCB), elevated pretreatment plasma levels of these markers might be useful for prediction of outcome [18]. Indeed, experimental data indicate that elevated levels of interleukin 6 (IL-6), a pro-inflammatory pleiotropic cytokine, are associated with aggressive tumor behavior in UCB [19, 20]. Overexpression of IL6 and its soluble receptor (IL6sR) has been reported in several malignancies, including prostate cancer and UCB [21–23]. In UCB, Andrews et al. reported an association of preoperative blood levels of IL6 and IL6sR on oncological survival outcomes in UCB, but their study was limited by their single-center nature, small sample sizes and lack of advanced statistical analyses [17, 23].

To externally validate the relationship of preoperative plasma IL-6 and IL-6sR levels with established features of bladder cancer invasion, metastasis and survival outcomes, we studied a large consecutive cohort of patients with non-metastatic advanced UCB treated with RC and pelvic lymphadenectomy. We hypothesized that patients with non-metastatic advanced UCB harboring occult metastases would have elevated levels of plasma IL-6 and IL-6sR which would be associated with features of biologically and clinically aggressive disease as well as poor survival despite effective local disease control. Our aim was to identify patients who are most likely to benefit from an intensified perioperative systemic therapy [24]. Beyond multivariable modeling, we used predictive accuracy testing and decision curve analysis (DCA) to assess real-world clinical utility of preoperative blood levels of IL6 and IL6sR as biomarkers.

## Methods

### Patients selection

All procedures described in the present study were undertaken with the approval and oversight of the Institutional Review Board for the Protection of Human Subjects. This study is retrospective analysis of prospectively collected consecutive cohort of patients who were treated with RC for non-metastatic UCB at two medical institutions. The extent of lymphadenectomy and choice of urinary diversion were at the surgeon’s discretion. Patients with any concomitant secondary malignancy, concomitant upper urinary tract carcinoma or missing data were excluded. No patient received neoadjuvant chemotherapy or radiotherapy. Adjuvant chemotherapy was administered to 167 patients (16.1%) at the clinicians' discretion based on tumor stage and overall health status. No patient received adjuvant radiotherapy.

### Biomarker measurements

Preoperative serum and plasma samples were collected typically on the morning of the day of surgery after an overnight fast. Specimen collection and measurement have been described in detail previously [25]. Briefly, blood was collected into Vacutainer CPT 8-ml tubes containing 0.1 ml of 1 m sodium citrate (Becton Dickinson, Franklin Lakes, NJ) and centrifuged at room temperature for 20 min at 1500 × *g*. The top layer corresponding to plasma was decanted using sterile transfer pipettes and immediately frozen and stored at − 80 °C in polypropylene cryopreservation vials (NalgeNunc, Rochester, NY). For quantitative measurements of IL-6 and IL-6sR levels, we used quantitative immunoassays (R&D Systems, Minneapolis, MN). Every sample was run in duplicate, and the mean was used. Differences between the two measurements for IL-6 and IL-6sR were minimal (intra-assay precision coefficients of variation: 5.2 ± 3.1, and 3.6 ± 2.9%, respectively).

### Pathological review and follow-up

All surgical specimens were processed according to standard pathological procedures as previously described [7]. All cases were histologically confirmed urothelial carcinoma of the bladder with only minor secondary variant components, if any. Genitourinary pathologists assigned tumor grade according to the 1973 WHO grading system. Pathologic stage was reassigned according to the 2002 American Joint Committee on Cancer TNM staging system. The presence of concomitant carcinoma in situ (CIS) was defined as the presence of CIS in conjunction with another tumor other than CIS [26]. Pelvic lymph nodes were examined grossly, and all lymphoid tissue was submitted for histological examination. Positive soft tissue surgical margin was defined as the presence of tumor at inked areas of soft tissue on the RC specimen [5]. Urethral or ureteral margins were not considered as soft tissue surgical margins. Lymphovascular invasion was defined as the unequivocal presence of tumor cells within an endothelium-lined space without underlying muscular walls [27].

Clinical and radiological follow-up was performed in accordance with institutional protocols and current guidelines. Routine follow-up usually included physical examination, radiological imaging and urine cytology every three months for two years. Between the second and the fifth year, follow-up was performed semiannually. Afterwards, in most cases, an annual follow-up was performed. Tumor recurrence was defined as the occurrence of locoregional recurrence or distant metastasis on radiological imaging. Cause of death was abstracted from medical charts and/or from death certificates [28].

### Statistical analysis

Report of categorical variables included frequencies and proportions. Reporting of continuous coded variables focused on medians and interquartile ranges (IQR). With respect to preoperative plasma levels of IL6 and IL6sR, which were treated as continuous variables, group comparisons were performed using the Mann–Whitney U tests, Kruskal–Wallis tests or calculation of Spearman's rank correlation coefficient (*r*) and subsequent significance testing, as appropriate. For analyses that required group classification, such as Kaplan–Meier survival curves or 5-year survival rates, stratification (low vs. high) was performed using median plasma levels of IL6 and IL6sR.

Binominal logistic regression analysis was performed for evaluating the association of preoperative plasma levels of IL6 and IL6sR with lymph node metastasis, ≥ pT3 disease or any non-organ confined disease (NOCD, defined as ≥ pT3 disease and/or lymph node metastasis). The area under the curve (AUC) of receiver operating characteristics (ROC) curves was calculated to determine the predictive accuracy of multiple logistic regression models. DeLong’s test was used to test for statistical significance between different AUCs. Association between preoperative IL6 and IL6sR with recurrence-free survival (RFS), cancer-specific survival (CSS) and overall survival (OS) was assessed in univariable and multivariable Cox regression models. Clinical and pathological tumor grade was excluded as a variable for all predictive models, since virtually all RC patients had high-grade UCB. Separate Cox regression models that featured either preoperative clinical variables or postoperative histopathological variables were created. The discriminative ability of these models after inclusion of IL6 and/or IL6sR was tested using Harrel’s concordance index (C-index). The additional clinical net benefit of both markers was also evaluated using decision curve analysis (DCA)[29]. Again, separate reference models that represented either the pre- or postoperative setting were created, to which IL6 and/or IL6sR were included in order to assess the additional predictive value of each biomarker. All reported p-values were two-sided, and statistical significance was set at 0.05. All statistical analyses were performed using R version 3.6.3.

## Results

### Association with clinicopathologic features

A total of 1,036 patients were included in the analysis. The median age of the entire cohort was 66.5 years (IQR 59.7–72.7). Median plasma levels of IL6 and IL6sR were significantly higher among patients with adverse pathologic features such as lymphovascular invasion, lymph node metastasis and advanced pathologic tumor stage (*p*-values < 0.05, Table 1). There was no relevant correlation between age and IL6 or IL6sR plasma levels (*r* = 0.05, *p* = 0.08 and *r* = 0.1, *p* < 0.001, respectively). There was also no correlation between plasma levels of both biomarkers (*r* = 0.05, *p* = 0.13) or between the number of lymph nodes removed and either IL6 (*r* = 0.005, *p* = 0.86) or IL6sR (*r* = 0.11, *p* = 0.57).

**Table 1:** Association of median preoperative plasma levels of il6 and il6sr with respect to clinicopathologic characteristics in 1036 patients treated with radical cystectomy for urothelial carcinoma of the bladder

Variable		n (%)	median plasma IL6 (IQR) pg/mL	*p*	median plasma IL6SR (IQR) ng/mL	*p*
Overall		1036 (100)	2.76 (2.12–3.99)		25.83 (20.6–30.64)	
Age (stratified by median age)	< 66.5 years	517	2.72 (2.11–3.92)	0.25	25.4 (20.4–30.3)	0.31
	> 66.5 years	519	2.77 (2.13–4.19)		26.4 (20.7–31.1)	
Sex	Male	814 (78.6)	2.76 (2.13–3.98)	0.89	25.85 (20.6–30.4)	0.65
	Female	222 (21.4)	2.75 (2.06–4.10)		25.81 (20.5–31.38)	
Blood transfusion	Yes	268 (25.9)	2.91 (2.22–4.67)	**0.018**	26.93 (21.28–32.2)	**0.02**
	No	768 (74.1)	2.69 (2.09–3.82)		25.4 (20.3–30.32)	
Thrombocytosis	Yes	113 (10.9)	2.77 (2.09–4.0)	0.41	26.4 (20.5–31.23)	0.45
	No	923 (89.1)	2.29 (2.71–4.0)		25.8 (20.6–30.45)	
Clinical tumor grade	Grade 2	6 (0.6)	2.89 (2.72–3.14)	0.6	21.3 (19.0–33.4)	0.61
	Grade 3	1022 (98.6)	2.74 (2.10–3.98)		25.88 (20.6–30.6)	
Clinical tumor stage	cTa	23 (2.2)	2.71 (2–3.58)	0.44	23.7 (19.9–28.44)	0.11
	cTis	105 (10.1)	2.93 (2.23–4.69)		23.6 (20.2–29.2)	
	cT1	336 (32.4)	2.65 (2.03–3.8)		26.15 (20.9–31.1)	
	cT2	498 (48.1)	2.77 (2.1–4.16)		26.28 (20.6–30.88)	
	cT3	38 (3.7)	2.84 (2.11–3.43)		29.2 (20.61–32.6)	
	cT4	29 (2.8)	2.83 (2.33–3.92)		25.2 (21.1–29)	
Pathological tumor grade	Grade 1	62 (6.0)	2.43 (2.11–3.01)	0.055	23.85 (18.02–30.2)	**0.011**
	Grade 2	11 (1.1)	2.33 (2.13–2.82)		20.6 (19.7–22.4)	
	Grade 3	963 (93.0)	2.79 (2.13–4.09)		26 (20.7–30.8)	
Pathological tumor stage	pT0	62 (6.0)	2.43 (2.12–3.01)	** < 0.001**	23.85(18.02- 30.2)	** < 0.001**
	pTa	22 (2.1)	2.46 (2.32–3.11)		20.6 (19.33–23.58)	
	pTis	131 (12.6)	2.83 (2.21–3.9)		23.7 (20.35–27.9)	
	pT1	162 (15.6)	2.47 (1.91–3.33)		26.85 (21.73–30.14)	
	pT2	248 (23.9)	2.67 (1.93–3.69)		25.3 (20.3–30.23)	
	pT3	281 (27.1)	2.93 (2.22–4.85)		27.7 (22.4–32.56)	
	pT4	130 (12.5)	3.04 (2.33–4.74)		26.49 (20.53–32.58)	
Upstaged to ≥ pT3 disease	Yes	357 (34.7)	2.98 (2.23–4.87)	** < 0.001**	27.6 (22.2–32.7)	** < 0.001**
	No	672 (65.3)	2.65 (2.02–3.56)		25 (20.3–29.8)	
Soft tissue surgical margin	Positive	95 (9.2)	2.83 (2.26–4.43)	0.09	28.3 (24.25–34.85)	**0.001**
	Negative	941 (90.8)	2.73 (2.11–3.97)		25.5 (20.4–30.37)	
Lymphovascular invasion	Positive	295 (28.5)	2.96 (2.28–4.74)	** < 0.001**	26.4 (21.4–31.8)	**0.042**
	Negative	741 (71.5)	2.65 (2.02–3.69)		25.7 (20.4–30.1)	
Concomitant Carcinoma in situ	Yes	572 (55.2)	2.81 (2.13–3.97)	0.74	25.33 (20.4–30.1	0.08
	No	464 (44.8)	2.69 (2.12–4.13)		26.43 (20.98–31.25)	
Lymph node metastasis	Yes	263 (25.4)	3.21 (2.35–4.8)	** < 0.001**	27.57 (22.19–32.05)	** < 0.001**
	No	773 (74.6)	2.63 (1.95–3.69)		25.3 (20.3–30.1)	
Use of adjuvant chemotherapy	Yes	167 (16.1)	2.99 (2.27–4.31	**0.018**	28.1 (21.84–32.35)	**0.005**
	No	869 (83.9)	2.7 (2.09–3.92)		25.5 (20.4–30.2)	
Recurrence	Yes	335 (32.3)	3.15 (2.32–5.04	** < 0.001**	30.6 (25.65–37.3)	** < 0.001**
	No	701 (67.7)	2.62 (1.94–3.59)		23.7 (20.00–28.2)	
Death of bladder cancer	Yes	303(29.2)	3.11 (2.32–5.0)	** < 0.001**	31.0 (26.35–37.4)	** < 0.001**
	No	733 (70.8)	2.64 (1.96–3.66)		23.7 (20.0–28.3)	
Death of any cause	Yes	564 (54.4)	2.77 (2.22–3.98)	0.29	27.90 (22.48–34.28)	** < 0.001**
	No	472 (45.6)	2.73 (2.03–3.99)		23.63 (20.0–28.1)	

Bold mean that result is significant, statistical significance was set at* p* < 0.05

**Fig. 1:**
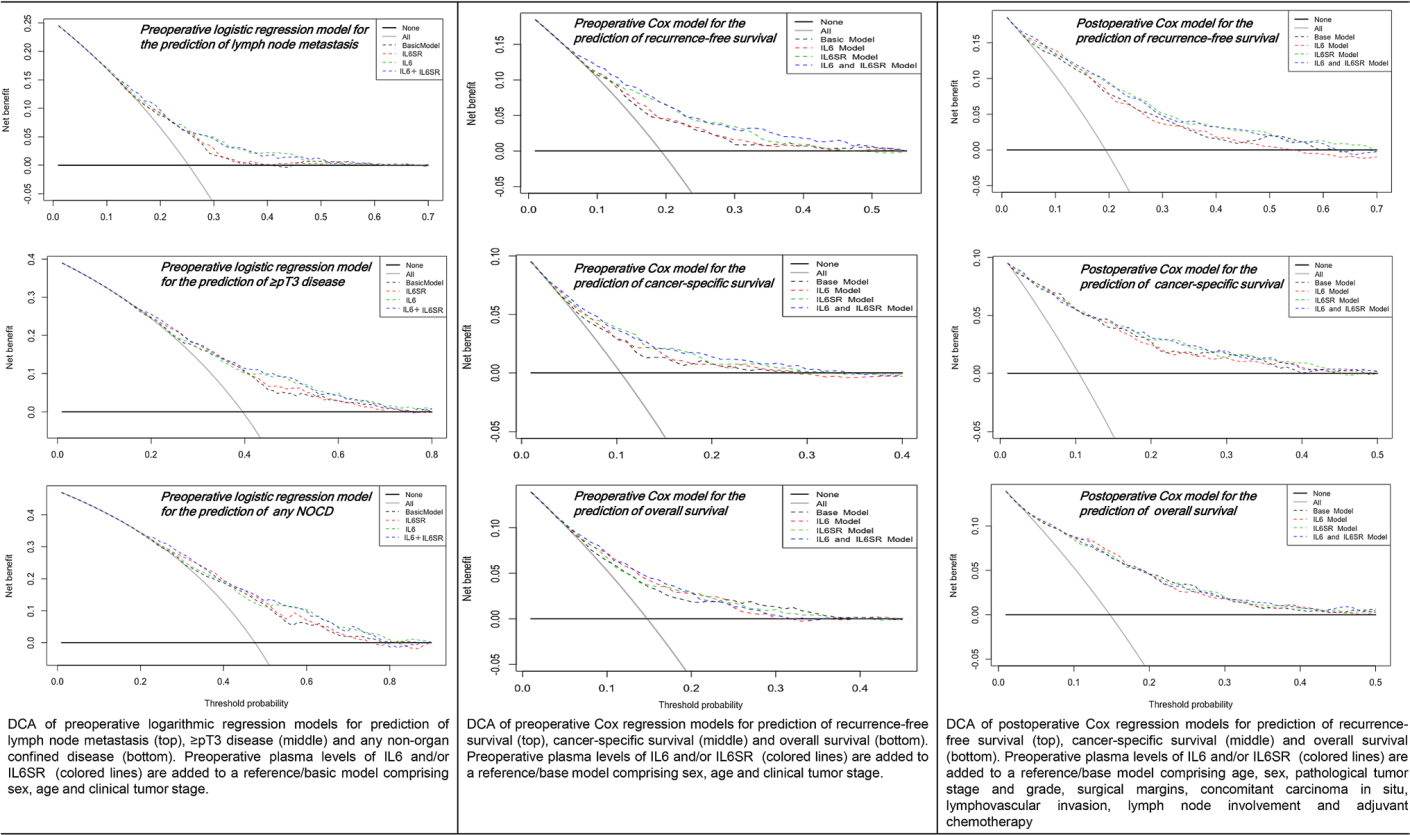
Decision curve analysis (dca) for the additional net benefit of il6 and il6sr based on separate reference models for the outcome prediction in 1,036 patients treated with radical cystectomy for urothelial carcinoma of the bladder. *Description* the x-axis is the threshold probabilities. The y-axis measures the net benefit which is calculated by adding the true positives and subtracting the false-positives. The horizontal line representing the x-axis assumes that no patients experiences the specified event whereas the grey line assumes that all patients will experience the specified event at a specific threshold probability. The dashed black line represents the net benefit of a basic reference model which was fitted using above mentioned variables. The dashed colored lines represent the net benefit of the same reference model which also includes the preoperative IL6 and IL6SR plasma levels as a variable

On multivariable logistic regression modeling, elevated preoperative plasma levels of both IL6 and IL6sR were significantly associated with an increased risk of ≥ pT3 disease, lymph node metastasis and any NOCD (*p*-values < 0.03, Table 2). ROC curve analysis showed that the addition of preoperative plasma levels of IL6 and IL6sR to a reference model comprising age, sex and clinical tumor stage significantly improved the discriminating ability for prediction of lymph node metastasis (5%, *p* < 0.001), ≥ pT3 disease (4%, *p* < 0.001) and any NOCD (4%, *p* < 0.001, Table 2). There were no significant differences between preoperative plasma levels of IL6 and IL6sR with respect to change of AUC. The highest values were achieved through addition of both markers. On DCA for prediction of ≥ pT3 disease, lymph node metastasis and any NOCD, only the addition of pretreatment plasma levels of IL6 to the previously described reference model resulted in a slight improvement of the clinical net benefit for prediction of lymph node metastasis, ≥ pT3 disease and any NOCD (Fig. 1).

**Table 2:** Multivariable logistic regression models for the prediction of lymph node metastasis, ≥ pt3 disease and any non-organ confined disease in 1,029 patients treated with radical cystectomy for urothelial carcinoma of the bladder

Variable	N *(%)*	Lymph node metastasis			≥ pT3 disease			Any non-organ confined disease		
		Odds ratio	95% CI	*p*	Odds ratio	95% CI	*p*	Odds ratio	95% CI	*p*
**IL6sR**	**1029** *(100)*	1.02	1.01–1.04	**0.03**	1.03	1.01–1.05	** < 0.001**	1.04	1.02–1.06	** < 0.001**
**IL6**	**1029** *(100)*	1.3	1.19–1.43	** < 0.001**	1.25	1.15–1.37	** < 0.001**	1.28	1.17–1.4	** < 0.001**
**Age**	**1029** *(100)*	0.99	0.98–1.01	0.43	1.02	1.01–1.04	** < 0.001**	1.02	1–1.03	** < 0.001**
**Male sex** *(Reference: female)*	**810** *(78.1)*	0.71	0.51–1.01	0.05	0.97	0.7–1.35	0.9	0.88	0.63–1.21	**0.008**
**Clinical tumor stage cT2** *(Reference: cT0/cTa/cTis/cT1)*	**498** *(48.4)*	2.5	1.83–3.46	** < 0.001**	2.78	2.1–3.69	** < 0.001**	3.09	2.35–4.07	** < 0.001**
**Clinical tumor stage ≥ cT3** *(Reference: cT0/cTa/cTis/cT1)*	**67** *(6.51)*	3.69	2.1–6.45	** < 0.001**	9.67	5.4–18.1	** < 0.001**	9.03	4.92–17.6	** < 0.001**
AUC preoperative reference model (*Age, Sex, clinical tumor Stage)*		**63%**			**67%**			**68%**		
AUC IL6sR + preoperative reference Model		**64%**			**69%**			**70%**		
AUC IL6 + preoperative reference model		**68%**			**70%**			**70%**		
AUC IL6sR and IL6 + preoperative reference model		**68%**			**71%**			**72%**		

Bold mean that result is significant, statistical significance was set at* p* < 0.05

### Association of survival outcomes with preoperative clinical variables

**Fig. 2:**
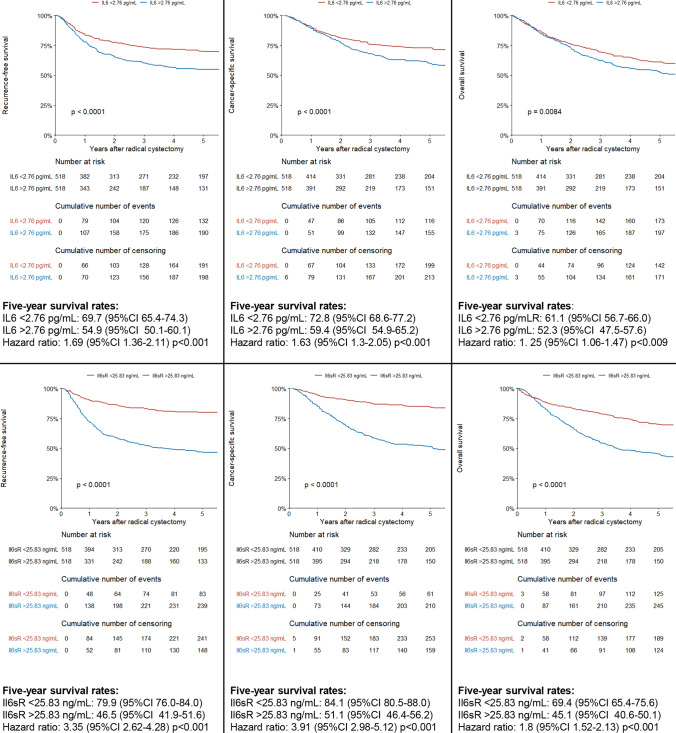
Kaplan–Meier curves, pairwise log rank tests and five-year survival analysis with respect to recurrence-free survival (left column); cancer-specific survival (middle column) and overall survival (right column) stratified by the preoperative median interleukin-6 (il6) plasma level (first row) and the interleukin-6 soluble receptor (il6sr) plasma level (second row) in 1,036 patients treated with radical cystectomy for urothelial carcinoma of the bladder

Median follow-up of patients alive was 37 months (IQR 14.5–108.5). The 5-year estimates for RFS, CSS and OS were 62.5% (95%CI 59.2–66%), 66% (95%CI 63.3 -70%) and 57% (95%CI 53.6–60.5%), respectively. Patients who experienced disease recurrence or died of UCB had significantly higher median pretreatment plasma levels of IL6 and IL6sR (*p* < 0.001). Patients who died of any cause also had significant higher median pretreatment plasma levels of IL6sR (*p* < 0.001), but not IL6 (*p* = 0.29). When stratified by median IL6 and IL6SR levels, patients with elevated plasma levels had significant worse survival outcomes with respect to RFS, CSS and OS (Fig. 2).

In a multivariable Cox regression model that included established preoperative available variables (age, sex and clinical tumor stage), higher pretreatment plasma levels of both IL6 and IL6sR were independently associated with worse RFS, CSS and OS (Table 3). The addition of preoperative plasma levels of both IL6 and IL6sR improved the C-indices of the same model for early prediction of RFS (11.4%), CSS (10.1%) and OS (4.4%). On DCA, only the addition of pretreatment IL6sR plasma levels to the same Cox model slightly improved the clinical net benefit of the model for early prediction of RFS and CSS between a threshold probability of 10–40%. There was no improvement for the prediction of OS across any threshold probability (Fig. 1).

**Table 3:** Separate pre- and postoperative multivariable cox regression models and c-index values for the prediction of recurrence-free survival, cancer-specific survival and overall survival in 1036 patients treated with radical cystectomy for urothelial carcinoma of the bladder

	Variable	N *(%)*	Recurrence-free survival			Cancer-specific survival			Overall survival		
			Hazard ratio	95% CI	*p*	Hazard ratio	95% CI	*p*	Hazard ratio	95% CI	*p*
Preoperative mode**l**	**IL6sR**	**1029** *(100)*	1.04	1.03–1.05	** < 0.001**	1.04	1.04–1.05	** < 0.001**	1.02	1.02–1.03	** < 0.001**
	**IL6**	**1029** *(100)*	1.32	1.23–1.41	** < 0.001**	1.33	1.24–1.42	** < 0.001**	1.2	1.13–1.27	** < 0.001**
	**Age**	**1029** *(100)*	1.01	1.0–1.02	0.13	1.01	1.0–1.03	**0.036**	1.05	1.03–1.06	** < 0.001**
	**Male sex** *(Reference: female)*	**810** *(78.1)*	0.69	0.54–0.88	**0.003**	0.66	0.51–0.85	**0.001**	0.75	0.63–0.93	**0.008**
	**Clinical tumor stage cT2** *(Reference: cT0/cTa/cTis/cT1)*	**498** *(48.4)*	1.8	1.42–2.29	** < 0.001**	1.99	1.54–2.57	** < 0.001**	1.65	1.38–1.97	** < 0.001**
	**Clinical tumor stage ≥ cT3** *(Reference: cT0/cTa/cTis/cT1)*	**67** *(6.51)*	2.31	1.53–3.49	** < 0.001**	2.62	1.71–4.01	** < 0.001**	1.93	1.4–2.66	** < 0.001**
	C-index preoperative reference model (*Age, Sex, Clinical tumor stage)*				**0.61**			**0.63**			**0.64**
	C-index IL6sR + preoperative reference Model				**0.69**			**0.72**			**0.66**
	C-index IL6 + preoperative reference model				**0.66**			**0.67**			**0.66**
	C-index IL6sR and IL6 + preoperative reference model				**0.72**			**0.73**			**0.68**
Postoperative model	**IL6sR**	**1036** *(100)*	1.04	1.03–1.05	** < 0.001**	1.05	1.04–1.05	** < 0.001**	1.02	1.02–1.03	** < 0.001**
	**IL6**	**1036** *(100)*	1.21	1.13–1.30	** < 0.001**	1.21	1.13–1.3	** < 0.001**	1.12	1.06–1.19	** < 0.001**
	**Age**	**1036** *(100)*	1	0.99–1.02	0.53	1.01	1–1.02	0.084	1.04	1.03–1.05	** < 0.001**
	**Male sex** *(Reference: female)*	**814** *(78.6)*	0.69	0.54–0.89	**0.004**	0.68	0.52–0.88	**0.004**	0.75	0.61–0.91	**0.004**
	**Pathological tumor stage pT2** *(Reference: pT0/pTa/pTis/pT1)*	**248** *(23.9)*	1.65	1.14–2.4	**0.008**	1.68	1.13–2.5	**0.01**	1.51	1.18–1.92	**0.001**
	**Pathological tumor stage ≥ pT3** *(Reference: pT0/pTa/pTis/pT1)*	**411** *(39.7)*	3.26	2.31–4.6	** < 0.001**	3.28	2.27–4.72	** < 0.001**	2.59	2.03–3.31	** < 0.001**
	**Lymphovascular invasion***(Reference: negative)*	**295** *(28.5)*	1.34	1.05–1.7	**0.02**	1.46	1.14–1.88	**0.003**	1.15	0.95–1.4	0.15
	**Positive soft tissue surgical margins** *(Reference: negative)*	**95** *(9.17)*	1.44	1.05–1.97	**0.02**	1.51	1.09–2.09	**0.012**	1.14	0.86–1.52	0.35
	**Concomitant CIS** *(Reference: negative)*	**572** *(55.2)*	1.01	0.81–1.27	0.9	0.93	0.73–1.18	0.55	1.02	0.86–1.22	0.8
	**Lymph node metastasis** *(Reference: negative)*	**264** *(25.5)*	2.45	1.9–3.17	** < 0.001**	2.46	1.88–3.22	** < 0.001**	2	1.62–2.47	** < 0.001**
	**Adjuvant chemotherapy** *(Reference: no)*	**167** *(16.1)*	0.79	0.6.1.04	0.09	0.89	0.67–1.18	0.42	0.83	0.66–1.05	0.11
	C-index postoperative reference model (*Age, Sex, Pathological Tumor Stage, Lymphovascular Invasion, Surgical Margin, Concomitant Cis and Lymph Node Involvement)*				**0.75**			**0.78**			**0.73**
	C-index IL6sR + postoperative reference Model				**0.78**			**0.80**			**0.74**
	C-index IL6 + postoperative reference model				**0.77**			**0.79**			**0.73**
	C-index IL6sR and IL6 + postoperative reference model				**0.79**			**0.81**			**0.74**

Bold mean that result is significant, statistical significance was set at* p* < 0.05

### Association of survival outcomes with postoperative histopathological variables

In a multivariable Cox regression model that focused on established postoperative variables, higher pretreatment plasma levels of both IL6 and IL6sR remained independently associated with worse RFS, CSS and OS (Table 3). The addition of preoperative plasma levels of both IL6 and IL6sR to the same prognostic model slightly improved the C-indices for prediction of RFS (4%), CSS (3.6%) and OS (1.5%). On DCA, only the addition of preoperative plasma levels of IL6sR slightly improved the clinical net benefit of the model for prediction of RFS and CSS between a threshold probability of 20–50%. Again, there was no improvement for the prediction of OS across any threshold probability (Fig. 1).

## Discussion

Despite additional studies that have added to our knowledge about molecular markers in UCB, no biomarker is used for individualized treatment recommendations, except urine cytology [11]. This is largely due to the lack of external validation in large, multicenter studies. In order to conclusively assess the potential predictive value of plasma levels of IL6 and IL6sR, we performed an external validation of its predictive value in an independent, large, multi-institutional cohort of patients who were treated with RC for UCB [17, 30, 31]. We were able to validate and extend our previous findings that elevated preoperative plasma levels of both biomarkers are independent predictors of lymph node metastasis and ≥ pT3 disease on logistic regression analyses [23]. Therefore, both biomarkers demonstrated the potential to identify patients with adverse pathologic features associated with aggressive biological and clinical behavior. These biomarkers, as part of a panel, could help in identifying those patients who may benefit from intensified/multimodal perioperative systemic therapy. Furthermore, we confirmed the independent association of elevated preoperative IL6 and IL6sR with worse survival outcomes.

It is well documented that inflammation is associated with the development and progression of cancer. IL-6 and IL6SR are produced by various normal cells, but also tumor-infiltrating immune cells and tumor cells themselves. It has therefore previously been concluded that increased systemic IL-6 levels are a result of local tumor production. Indeed, Andrews et al. found that plasma levels of IL-6 and IL-6sR are associated with tumor stage and metastases and are strong independent predictors of disease recurrence and disease-specific survival [23]. However, the question of whether one or both of these biomarkers can improve the prognostic use of established predictors of cancer outcome requires more than the conventional multivariable analyses in small cohorts, as previously performed by Andrews et al. [23]. It must be established that the use of a prognostic biomarker adds unique information that improves the performance of a predictive model constructed without the new biomarker by a statistically significant margin [17, 30]. Therefore, we tested whether preoperative plasma IL6 and/or IL6sR improved the accuracy of predictive models and whether they added net clinical benefit in the pre- and/or postoperative setting [23]. We found that the addition of preoperative IL6 and IL6sR to a reference model, which included only preoperatively available variables, significantly improved the discriminatory power for prediction of non-organ confined disease. Both biomarkers also demonstrated the ability to improve the performance of the same model for early prediction of RFS (+ 11.4%) and CSS (+ 10%), even though the overall discriminatory power of the model was only moderate. On DCA, the addition of IL6 slightly improved the clinical net benefit of the same model. Thus, preoperative IL6 and IL6sR can guide preoperative risk stratification through improved early outcome prognostication and may help improve patient selection for neoadjuvant systemic therapy [11–15, 24].

Both biomarkers also demonstrated the ability to improve outcome prediction if added to a model that consisted of established histopathological variables. In this postoperative setting, our prognostic model exhibited a high overall discriminatory power (C-Index of 0.79 and 0.81 for prediction of RFS and CSS, respectively). Such an accurate outcome prediction would allow for tailored therapy and thus improve patient care. However, even the addition of both biomarkers did not improve the discriminatory power for prediction of OS, suggesting that both are cancer-specific biomarkers. On DCA, however, the addition of preoperative IL6sR slightly improved the net benefit of this model for prediction of both RFS and CSS. Our findings warrant further validation of preoperative IL6 and IL6sR in the context of correlative biomarker assessment integrated in prospective clinical trials [30]. Furthermore, both carry other advantageous features of clinically beneficial biomarkers, as they are easily accessible, cost-effective and allow early outcome prediction [31]. Since IL6 and IL6sR are systemic inflammatory markers, they might prove especially useful in the prediction of response to immunotherapy. Targeting IL6 and IL6sR through monoclonal antibodies is being researched as a novel treatment strategy in UCB, and in this particular setting, plasma levels of IL6 and IL6sR could permit individualization of therapy [32]. In summary, our findings warrant the inclusion of these biomarkers into future predictive/prognostic models in order to increase the discriminatory power and allow a personalized medicine approach, as patients with elevated biomarker levels are more likely to harbor adverse pathological features and experience poor survival outcomes.

The main limitation of our study is its retrospective analysis. Also, only the pretreatment plasma levels of IL6 and IL6sR levels were assessed in this study. Confounding conditions, such as undiagnosed infectious diseases or unknown drug interaction, could potentially have affected plasma levels of IL6 and IL6sR. However, this would have weakened an existing potential association. Data on therapies before RC, which might also cause inflammation and alter levels of IL6 and IL6sR, such as intravesical BCG instillations, were unfortunately, unavailable. Due to the time of recruitment of this study, there is no information available on the predictive value of IL6 and IL6sR with respect to immunotherapies or neoadjuvant chemotherapy. The strength of this cohort is it purity in treatment allocation. Its weakness is that it does not reflect current treatment standards. Another limitation is that the short median follow-up with 37 months. However, we and others have shown that over two-thirds of patients experience disease recurrence after RC within 12 months and ≥ 90% in 24 months [33].

## Conclusions

We externally validated that elevated preoperative plasma levels of IL6 and IL6sR levels are associated with worse survival in an independent international large cohort of patients treated with RC for UCB. Moreover, both biomarkers hold potential in identifying patients with adverse pathologic features that may benefit from intensified/multimodal therapy. They also demonstrated the ability to improve the discriminatory power of current prognostic models and thus can help guide clinical decision making.

## Data Availability

The datasets generated during and/or analyzed during the current study are available from the corresponding author on reasonable request.

## Code availability

Throughout the manuscript, only basic and established statistical tests are applied. The codes are available on reasonable request.
